# High-Field Open versus Short-Bore Magnetic Resonance Imaging of the Spine: A Randomized Controlled Comparison of Image Quality

**DOI:** 10.1371/journal.pone.0083427

**Published:** 2013-12-31

**Authors:** Judith Enders, Matthias Rief, Elke Zimmermann, Patrick Asbach, Gerd Diederichs, Christoph Wetz, Eberhard Siebert, Moritz Wagner, Bernd Hamm, Marc Dewey

**Affiliations:** Department of Radiology, Charité, Medical School, Humboldt-Universität zu Berlin and Freie Universität Berlin, Germany; Charité University Medicine Berlin, Germany

## Abstract

**Background:**

The purpose of the present study was to compare the image quality of spinal magnetic resonance (MR) imaging performed on a high-field horizontal open versus a short-bore MR scanner in a randomized controlled study setup.

**Methods:**

Altogether, 93 (80% women, mean age 53) consecutive patients underwent spine imaging after random assignement to a 1-T horizontal open MR scanner with a vertical magnetic field or a 1.5-T short-bore MR scanner. This patient subset was part of a larger cohort. Image quality was assessed by determining qualitative parameters, signal-to-noise (SNR) and contrast-to-noise ratios (CNR), and quantitative contour sharpness.

**Results:**

The image quality parameters were higher for short-bore MR imaging. Regarding all sequences, the relative differences were 39% for the mean overall qualitative image quality, 53% for the mean SNR values, and 34–37% for the quantitative contour sharpness (*P*<0.0001). The CNR values were also higher for images obtained with the short-bore MR scanner. No sequence was of very poor (nondiagnostic) image quality. Scanning times were significantly longer for examinations performed on the open MR scanner (mean: 32±22 min versus 20±9 min; *P*<0.0001).

**Conclusions:**

In this randomized controlled comparison of spinal MR imaging with an open versus a short-bore scanner, short-bore MR imaging revealed considerably higher image quality with shorter scanning times.

**Trial Registration:**

ClinicalTrials.gov NCT00715806

## Introduction

Conventional magnetic resonance (MR) imaging is performed with the patient lying in a long, narrow tube [1]. Thus, its applicability can be limited, for example, in patients with claustrophobia or extreme obesity [2], [3], [4]. In recent years, MR scanners with specific patient-centered designs have been developed. Two promising approaches are short-bore and horizontal open configurations. Modern MR scanners with these configurations have already shown a potential for reducing claustrophobia and allowing imaging of extremely obese patients [2], [5], [6], [7], [8], [9]. Previous studies have also demonstrated improved patient acceptability of open-configuration MR scanners [5], [10], [11]. Besides patient preference, diagnostic performance and thus image quality is crucial for a comparison of different MR systems. An open scanner configuration might impair image quality due to a potentially larger inhomogeneity of a vertical magnetic field. Moreover, until recently, such systems operated at rather low field strengths [11], [12], [13]. However, there is no study directly comparing the image quality of horizontal and vertical magnetic field MR systems.

The objective of this analysis was thus to quantitatively and qualitatively compare the image quality of two high-field MR scanners, one with a short (1.5 m) and wide (0.6 m) bore and one with a horizontal open configuration, for spinal imaging. The patients included in the analysis were part of a larger cohort. All patients were at increased risk to suffer from cluastrophobia as the objective of this randomized controlled study was to compare patient-centered MR systems. To our knowledge, this is the first study to compare recent high-field MR scanners with patient-centered designs. The insights to be derived from this analysis are important to be able to define which further improvements in MR imaging technology might be necessary.

## Materials and Methods

### Ethics Statement

Approval was obtained from the institutional review board at Charité, Berlin. All patients gave written informed consent. This trial was conducted and is reported in accordance with the Declaration of Helsinki and the CONSORT guidelines for nonpharmacological randomized trials [14]. The supporting CONSORT checklist is available as supporting information; see Checklist S1. This trial has been registered in ClinicalTrials.gov (Identifier: NCT00715806). The main results have been published in PLoS ONE [8]. The detailed trial protocol has been published elsewhere [15], and is available as supporting information; see Protocol S1.

### Study Design

Between June 19, 2008 and August 14, 2009, we performed a prospective single-center parallel-group randomized controlled trial in 174 consecutive patients in a university hospital [8]. Follow-up was performed clinically until 7 months after randomization without another MR imaging session as reported in the *Journal* [8]. Inclusion criteria were a clinical indication for MR imaging of the head, spine, or shoulder, and a total mean score of at least 1.0 in the Claustrophobia Questionnaire (CLQ, score range: 0 to 4) [16]. Exclusion criteria were absolute or relative contraindications to MR imaging [17], body weight of more than 200 kg (due to safety restrictions of the MR tables), and age below 18 years [15].

**Figure 1:**
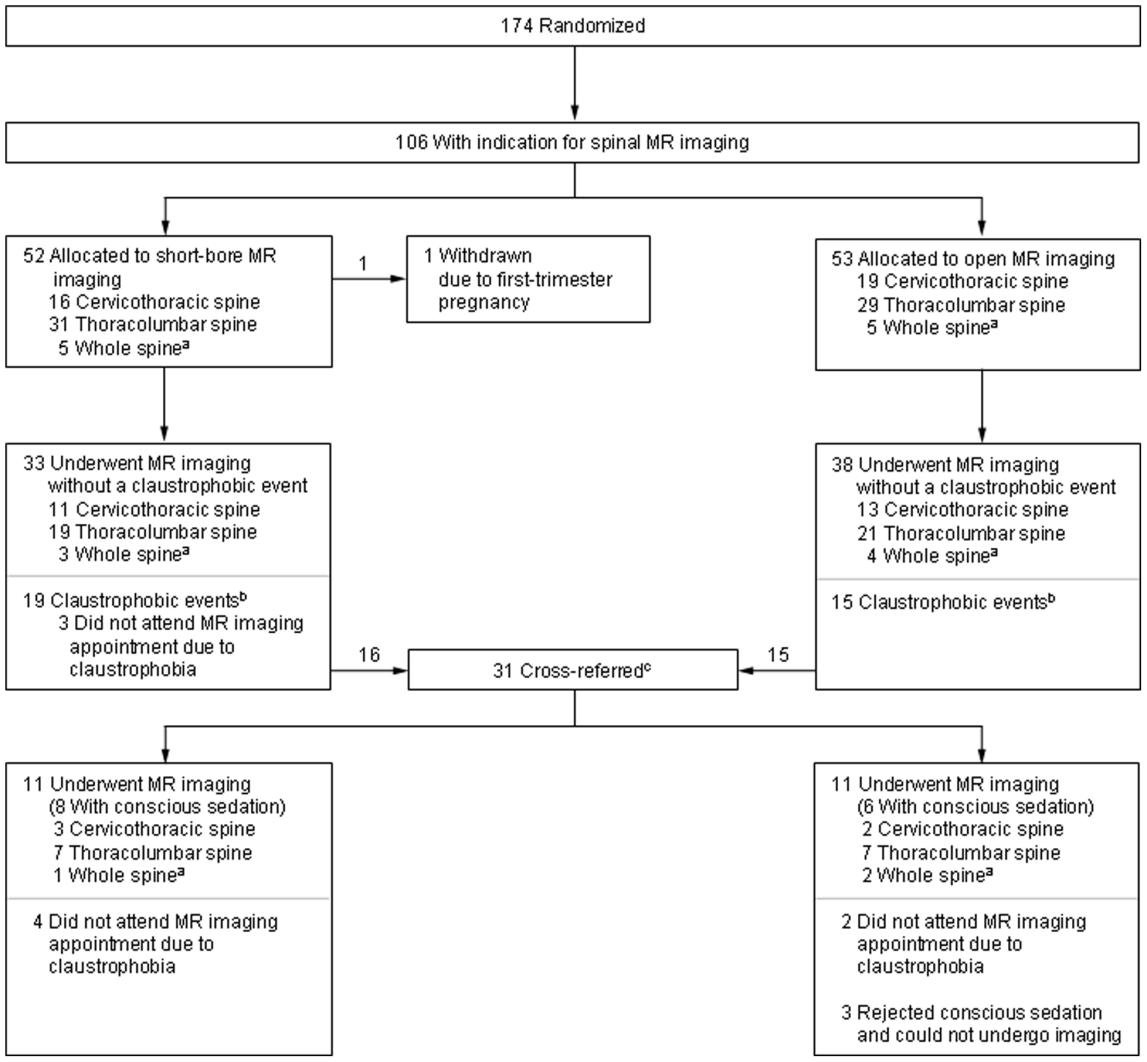
Randomization, Anatomical Regions, and Claustrophobic Events in the Study. Ninety-three of the 106 patients with a clinical indication for spinal MR imaging underwent an MR examination in our study. ^a^For the analysis, MR imaging of the whole spine was categorized as imaging of the cervicothoracic and the thoracolumbar spine. ^b^A claustrophobic event was defined as the inability of a patient to undergo MR imaging due to claustrophobia. All but one patient with a claustrophobic event rejected MR imaging before the examination started. The one patient aborted MR imaging on the short-bore MR scanner after acquisition of one sequence, which could thus not be included in the analysis. ^c^Patients were cross-referred for a second MR examination on the other scanner if they could not bear imaging on the first scanner in order to avoid the risks of conscious sedation. Only patients who could not undergo MR imaging in either of the two scanners received conscious sedation (IV midazolam) according to the American Society of Anesthesiology guideline [18]. Sedation was performed using IV midazolam (sedation success rate 100%, no adverse events). Examinations performed after cross-referral, with or without sedation, were included in the primary analysis.

Eligible patients were randomly assigned (computer-generated sequence) in a 1∶1 ratio to: 1) MR imaging in an open panoramic state-of-the-art scanner with a vertical magnetic field, 1-T field strength, up to 26 mT/m gradient strength, maximum acoustic noise of 150 dB(A), and an 0.45 m high and 1.6 m wide patient aperture (0.7 m wide patient table) (Panorama, Philips Medical Systems) [5], or 2) MR imaging in a short-bore state-of-the-art scanner with 1.5-T field strength, up to 45 mT/m gradient strength, 97% noise reduction to below 99 dB(A), and a conical wide (0.6 m) and short (1.5 m) bore (Magnetom Avanto, Siemens Medical Solutions) [2]. Randomization was not stratified and allocation was concealed (using sealed envelopes). Patients could not be blinded to the assigned study group due to the MR imaging setting. If patients did not complete imaging in their assigned MR scanner due to claustrophobia, they were cross-referred to imaging in the other scanner in order to avoid the risks of conscious sedation [15]. See Figure 1 for the flow of patients. Only patients who could not undergo MR imaging in either of the two scanners received conscious sedation (IV midazolam) according to the American Society of Anesthesiology guideline [18]. Examinations performed after cross-referral, with or without sedation, were included in the primary analysis but were also assessed separately in subgroup analyses.

The patients included were part of a lager cohort. In a previous analysis the frequency of claustrophobic events was assessed. An event was defined as the prevention of MR imaging due to claustrophobia [8]. Image quality comparison of MR imaging of the head and the shoulder will be the objective of future analyses because of differences in the MR imaging setting, including different coils and sequences, for these examinations.

### MR Imaging

MR imaging was performed in the supine position using phased-array coils. A large solenoid coil (ST body/spine XL) was used on the horinzontal open MR scanner, and the spine-array coils that are integrated into the table were used on the short-bore scanner. Patients were examined head-first for imaging of the cervicothoracic spine and imaging of the whole spine. For thoracolumbar spine imaging, a feet-first approach was used. The basic MR sequences acquired were sagittal T2- and T1-weighted, and axial T2-weighted sequences. Detailed information on the sequence parameters can be found in the trial protocol [15], see Protocol S1. In 15 examinations optional sequences were acquired (9 sagittal turbo inversion recovery and 12 T1-weighted sequences after contrast medium administration), which were not included in the analysis due to the small number. All sequences were confirmed by local application specialists, and the primary aim of the sequence setup was to obtain a voxel size and imaging time that is as similar as possible on both scanners. Parallel imaging techniques were not used because this would have affected quantitative image quality parameters such as signal-to-noise ratio (SNR) and contrast-to-noise ratio (CNR) [21]. The scan duration, defined as the time from the beginning of the first to the end of the last sequence, was documented. A custom-made questionnaire was used to track certain features of the MR procedure (e.g., pain, noise) [15].

### Image Analysis

The qualitative analysis was performed by two examiners in consensus, and the quantitative analysis was performed by one examiner on a workstation (Centricity PACS Workstation RA 1000, GE Healthcare) [15]. At the time of the analysis the examiners were blinded to the imaging technique and patient identity. The optimal viewing window-level parameters for each image were adjusted automatically by the examiners with a rectangular region of interest (ROI) positioned in a region including cerebrospinal fluid, disc tissue, and vertebral body, and standardized in size according to the anatomy. MR imaging of the whole spine was categorized as imaging of the cervicothoracic and the thoracolumbar spine. Imaging of the thoracic spine was performed using the MR protocol for cervicothoracic or thoracolumbar spine, depending on the indication.

Qualitative image analysis was performed via grading from 1 to 5. For the rating of contrast, contour sharpness, and overall image quality the scale was 1 = optimal, 2 = good, 3 = moderate, 4 = poor, and 5 = very poor (nondiagnostic). The scale for rating artifacts and noise was 1 = none, 2 = minimal, 3 = moderate, 4 = major, and 5 = nondiagnostic. Artifacts were classified as being due to motion, pulsation, metal, noise, or other [15].

Quantitative image analysis was performed by measurement of signal intensities (SI) in circular ROIs. They were placed on corresponding anatomical levels in areas without signal abnormalities and standardized in size according to the anatomy. Standard deviations (SD) of the ROIs were used to measure noise, as noise is known to vary across the field of view (FOV) when phased-array coils are used [22]. SNR and CNR were calculated as recently described [22], using the following formulas: SNR_tissue_ = SI_tissue_/SD_tissue_, CNR = SNR_tissue A_−SNR_tissue B_, (SNR_tissue A_>SNR_tissue B_). Moreover, contour sharpness was analyzed as recently described [23], using ImageJ open access software (http://rsbweb.nih.gov/ij/). For detailed information on the analysis see Figure S1. It was analyzed for the interface between corticospinal fluid and spinal cord as well as between corticospinal fluid and vertebral body/posterior longitudinal ligament.

### Statistical Analysis

The chi-squared test, the unpaired t-test, and the Fisher exact test were used as appropriate for categorical and continous variables. The nonparametric Mann-Whitney rank sum test was used in the qualitative analysis as these data were not normally distributed. Correlation analyses were performed with the Pearson correlation test. All tests were two-sided, and the level of significance was set at 5% (*P*<0.05). Statistical analyses were conducted using SPSS version 16.0 (Chicago, IL, US).

## Results

### Participants

Of 174 enrolled patients, 106 had a clinical indication for MR imaging of the spine: 35 cervicothoracic spine, 61 thoracolumbar spine, 10 whole spine. Of these 106 patients, 93 underwent MR imaging, and all were included in this analysis (Figure 1). Table 1 lists the baseline characteristics of the patients included. They were well matched between both groups. Eighty percent of the patients were women who have been shown to be more likely to suffer from claustrophobia [8]. The mean age was 53 (SD, 12; range, 27-88), and the mean body mass index (BMI) was 29 (SD, 7.5; range, 17.5–52). All patients were at increased risk to suffer from claustrophobia. The mean CLQ score was 2,35 (SD, 0.69) which is in accordance with other high-risk groups [16]. Moreover, 50.5% of the 93 patients had prior MR imaging which was prevented, aborted or performed with sedation due to claustrophobia. Indications for MR imaging included: radicular pain (n = 60), (non-motor) neurologic symptoms and/or deficits (n = 60), motor deficits (n = 28), history of prior surgery (n = 10), cauda equina syndrome (n = 4), suspicion of cancer (n = 3), and history of previous trauma (n = 2). Some patients had more than one clinical indication for MR imaging. All indications were appropriate according to American College of Radiology guidelines [8], [19], [20].

**Table 1:** Characteristics of the 93 Randomized Patients who Underwent Spinal MR Imaging.

	Short-Bore MR (n = 44)	Open MR (n = 49)	*P* Value
Female sex	35 (79.5)	40 (81.6)	.8
Age	53 (SD, 11.4)	53.1 (SD, 12.7)	.1
Age categories			.7
<30	0 (0)	1 (2)	
30 - <50	22 (50)	23 (46.9)	
50 - <70	16 (36.4)	20 (40.8)	
≥70	6 (13.6)	5 (10.2)	
Body height in cm	167.9 (SD, 8.6)	168.3 (SD, 10.3)	.8
Body weight in kg	79.6 (SD, 23.3)	84.9 (SD, 25.9)	.3
Body mass index (BMI)	28.1 (SD, 7.7)	29.7 (SD, 7.3)	.3
BMI categories			.5
<20	4 (9.1)	1 (2)	
20- <3	(54.5)	28 (57.1)	
30- <40	12 (27.3)	15 (30.6)	
≥40	4 (9.1)	5 (10.2)	
Maximum body circumference in cm	112.8 (SD, 17.3)	115.6 (SD, 14.4)	.4
Region of MR imaging			.9
Cervicothoracic spine	14 (31.8)	15 (30.6)	
Thoracolumbar spine	26 (59.1)	28 (57.1)	
Whole spine	4 (9.1)	6 (12.2)	
Claustrophobia Questionnaire mean value^a^	2.38 (SD, 0.75)	2.32 (SD, 0.65)	.7
State anxiety before MR imaging^b^	2.65 (SD, 0.56)	2.65 (SD, 0.68)	1

Data are number (%) or arithmetic mean (SD). Percentages may not total 100% because of rounding.

^a^ The Claustrophobia Questionnaire (CLQ) [16] consists of 26 items which assess two separate but related fears hypothesized to comprise claustrophobia: the fear of suffocation and the fear of restriction. For each of the 26 items of the CLQ, anxiety is rated on a scale from 0 (not at all anxious) to 4 (extremely anxious).

^b^ Directly before MR imaging, the State questionnaire of the Spielberger State-Trait Anxiety Inventory (STAI) [33] was used to assess patients' state anxiety. It consits of 20 items to be rated on a scale from 1 (almost never) to 4 (very much so).

### MR Imaging

In the 93 patients who underwent spinal MR imaging there were 48 examinations on the short-bore MR scanner and 55 examinations on the open MR scanner. 25 examinations were performed after cross-referral, 16 of them with conscious sedation (Figure 1, Table 2). Only one patient aborted MR imaging on the first scanner due to claustrophobia after one sequence, which was thus not included in the primary analysis. The other patients who were imaged after cross-referral rejected MR imaging on the first scanner before the examination had started (Figure 1). Thus, none of the patients was scanned on both scanners. Details of the MR imaging characteristics are listed in Table 2. Scanning times were significantly longer for open MR imaging and the perceived noise and pain levels were also higher, although statistically not significant, in patients who were examined with the open MR scanner [8].

**Table 2:** Characteristics of the MR Examinations.

	Short-Bore MR Group	Open MR	*P* Value
Number of MR examinations^a^	48	55	.9
Cervicothoracic spine	18 (37.5)	21 (38.2)	
Thoracolumbar spine	30 (62.5)	34 (61.8)	
Number of MR sequences			
T1w/T2w sagittal	48	55	.9
Cervicothoracic spine	18 (37.5)	21 (38.2)	
Thoracolumbar spine	30 (62.5)	34 (61.8)	
T2w axial^b^	68	76	.9
Cervicothoracic spine	19 (28)	22 (28.9)	
Thoracolumbar spine	49 (72)	54 (71.1)	
Scan duration (min)	19.8 (SD, 8.5)	31.7 (SD, 21.6)	.001
Cervicothoracic spine	19 (SD, 5.3)	30.1 (SD, 7.8)	.001
Thoracolumbar spine	17.5 (SD, 7.1)	25.8 (SD, 10.2)	.001
Whole spine	37 (SD, 6.8)	70 (SD, 42)	.2
Anxiety^c^	58.1 (SD, 33.9)	50.3 (SD, 30.6)	.3
Cervicothoracic spine	58.6 (SD, 34.9)	48.1 (SD, 30.1)	.4
Thoracolumbar spine	55.8 (SD, 32.8)	51.8 (SD, 31.9)	.7
Whole spine	71 (SD, 44.8)	49 (SD, 30.5)	.4
Noise^c^	57.5 (SD, 21.3)	65.9 (SD, 23.1)	.08
Cervicothoracic spine	52.2 (SD, 24)	62.5 (SD, 25)	.3
Thoracolumbar spine	58.5 (SD, 18.3)	71.6 (SD, 21.1)	.02
Whole spine	69.8 (SD, 29.2)	47.7 (SD, 18.6)	.2
Pain^c^	19.8 (SD, 27.5)	31 (SD, 32.5)	.08
Cervicothoracic spine	20 (SD, 29.7)	27.6 (SD, 28.7)	.5
Thoracolumbar spine	21.2 (SD, 28.3)	28.6 (SD, 33.8)	.4
Whole spine	10.5 (SD, 15.8)	51.2 (SD, 33.7)	.06

Data are number (%) or arithmetic mean (SD). Percentages may not total 100% because of rounding.

^a^ For the analysis, MR imaging of the whole spine was categorized as imaging of the cervicothoracic and the thoracolumbar spine.

^b^ The number of axial sequences and the segment of the vertebral column that was imaged with axial sequences were chosen according to the medical indication.

^c^ The pain, noise, and anxiety levels patients experienced during MR imaging were assessed directly after the scan using horizontal and nonmarked (0–100 mm) visual analogue scales.

Results of all examinations were of diagnostic image quality. The main findings included: disc protrusion (n = 58), disc extrusion (n = 44), degenerative spondylarthrosis (n = 45), neural foraminal narrowing (n = 42), nerve root compression/irritation (n = 35), spinal stenosis (n = 33), intervertebral osteochondrosis (n = 28), myelopathy/spinal cord lesions (n = 5), fracture (n = 4), metastasis (n = 2), spondylolisthesis (n = 2), and spondylodiscitis (n = 1).

### Qualitative Analysis

**Figure 2:**
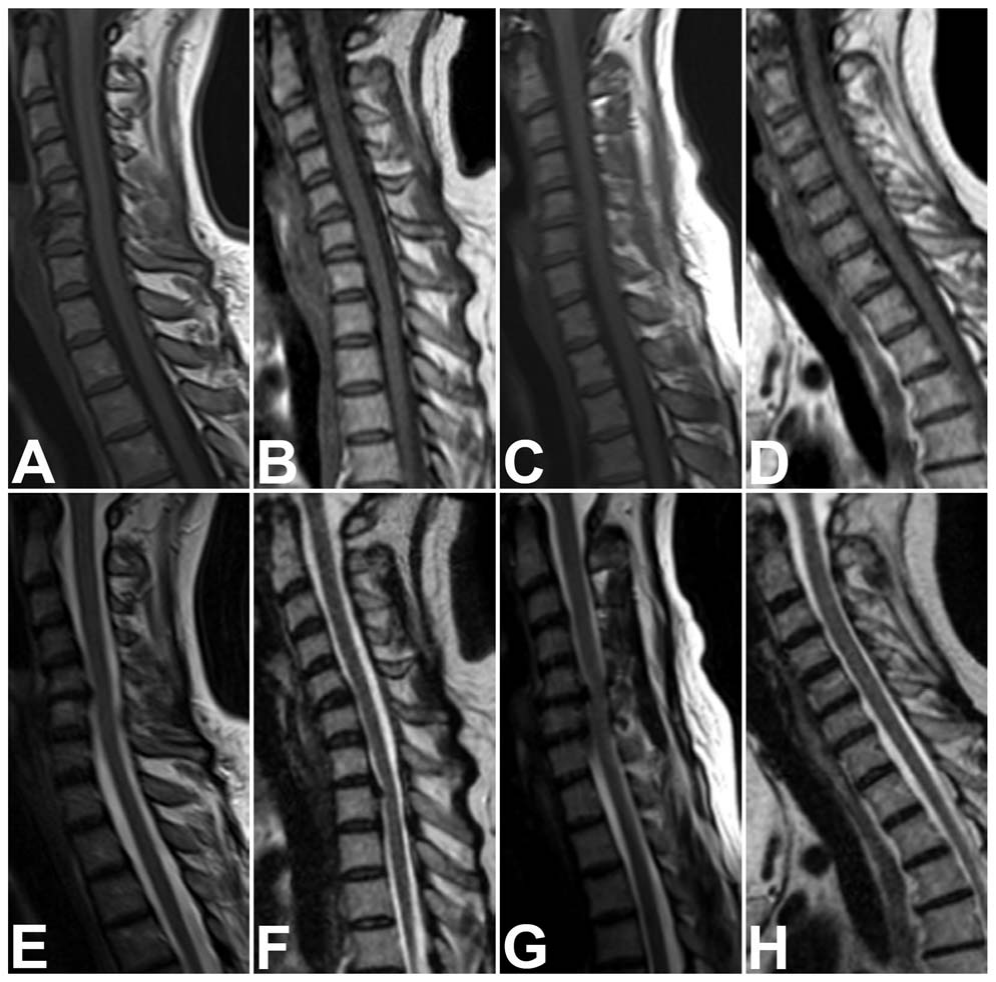
Representative Examples of Image Quality on Sagittal T1- and T2-Weighted Sequences of the Cervical Spine. Top row: sagittal T1-weighted turbo spin-echo sequences of the cervicothoracic spine of four patients, representing the range of qualitative image quality scores obtained with short-bore (A, C) and open MR imaging (B, D). Overall image quality ratings were: “optimal” for sequence A,“good” for B, “moderate” for C, and “poor” for D. No sequence was rated very poor (nondiagnostic). Bottom row: sagittal T2-weighted turbo spin-echo sequences of the cervicothoracic spine of the same four patients obtained with short-bore (E, G) and open MR imaging (F, H). Overall image quality ratings were: “good” for sequence E,“good” for F, “moderate” for G, and “good” for H. No sequence was rated very poor (nondiagnostic).

**Figure 3:**
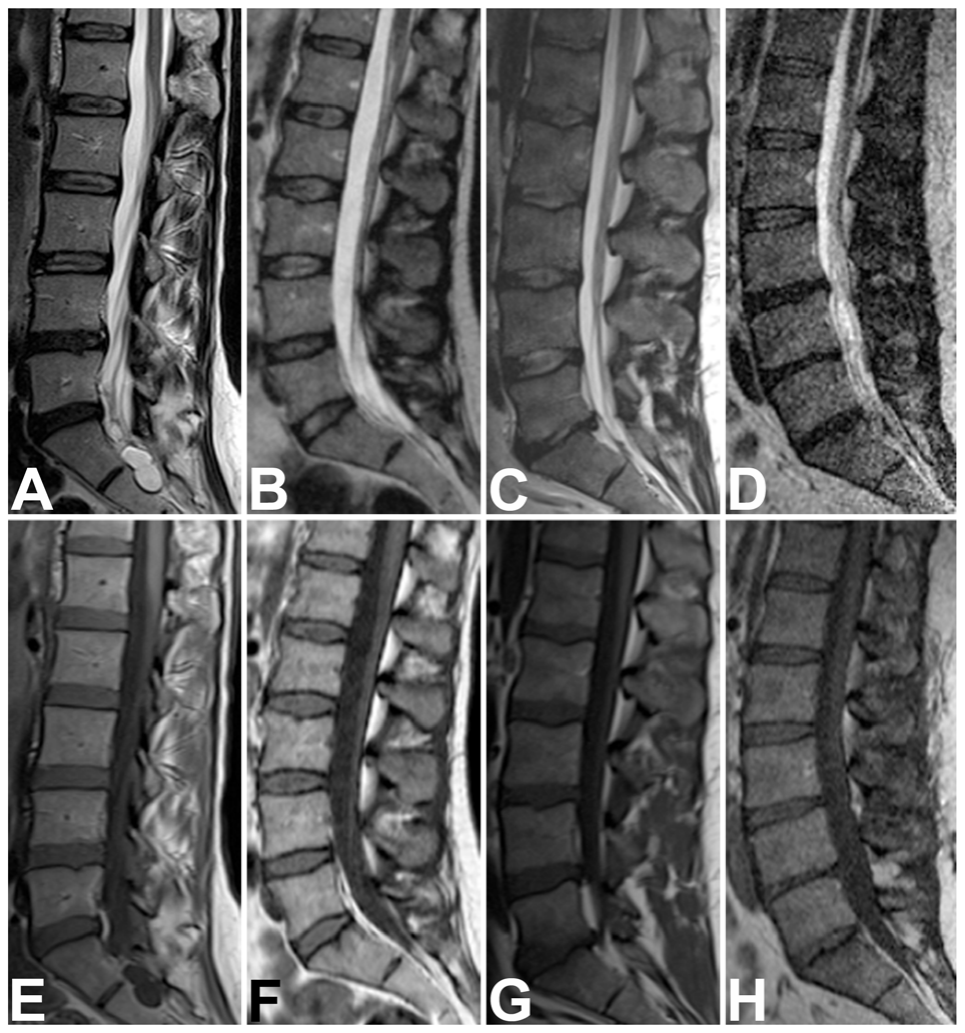
Representative Examples of Image Quality on Sagittal T2- and T1-Weighted Sequences of the Lumbar Spine. Top row: sagittal T2-weighted turbo spin-echo sequences of the lumbar spine of four patients, representing the range of qualitative image quality scores obtained with short-bore (A, C) and open MR imaging (B, D). Overall image quality ratings were: “optimal” for A,“good” for B, “moderate” for C, and “poor” for D. No sequence was rated very poor (nondiagnostic). Bottom row: sagittal T1-weighted turbo spin-echo sequences of the lumbar spine of the same four patients obtained with short-bore (E, G) and open MR imaging (F, H). Overall image quality ratings were: “good” for sequence E,“moderate” for F, “good” for G, and “poor” for H. No sequence was rated very poor (nondiagnostic).

**Figure 4:**
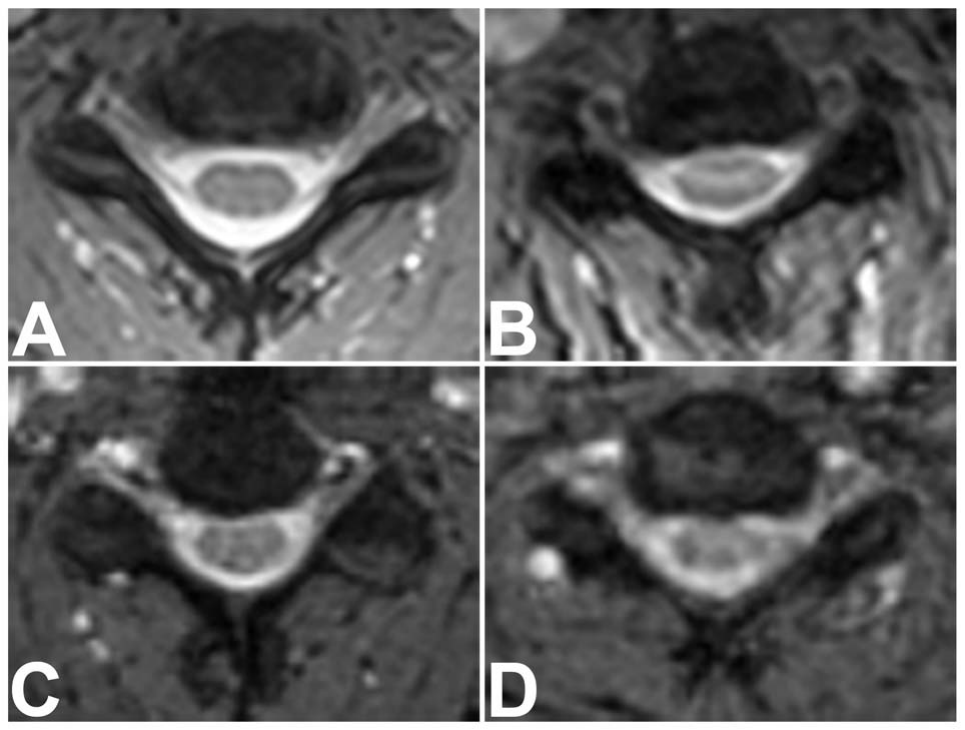
Representative Examples of Image Quality on Axial T2-Weighted Sequences of the Cervical Spine. Axial T2-weighted medic sequences of the cervical spine of four patients, representing the range of qualitative image quality scores obtained with short-bore (A, B) and open MR imaging (C, D). Overall image quality ratings were: “optimal” for sequence A,“good” for sequence B, “moderate” for C, and “poor” for D. No sequence was rated very poor (nondiagnostic).

**Figure 5:**
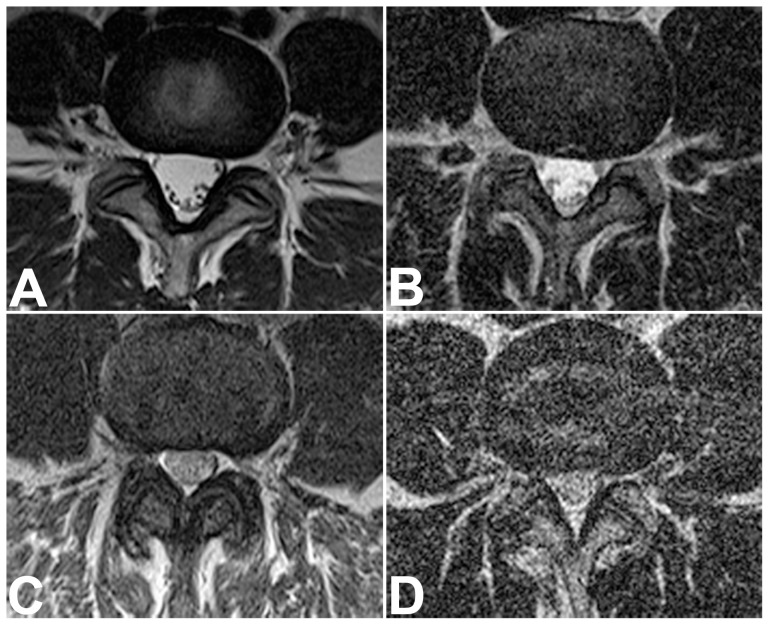
Representative Examples of Image Quality on Axial T2-weighted Sequences of the Thoracolumbar Spine. Axial T2-weighted sequences of the lumbar spine of four patients, representing the range of qualitative image quality scores obtained with short-bore (A, C) and open MR imaging (B, D). Overall image quality ratings were: “optimal” for sequence A,“good” for sequence B, “moderate” for C, and “poor” for D. No sequence was rated very poor (nondiagnostic).

Qualitative overall image quality of all available imaging sequences, assessed by two blinded examiners in consensus, was rated significantly higher for short-bore MR images than for open MR images (1.92 [SD, 0.74] versus 3.16 [SD, 0.77]; *P*<0.0001). No sequence was rated very poor (nondiagnostic). Thus image quality scores ranged from 1 (optimal) to 4 (poor). Table 3 shows the respective results computed separately for all qualitative parameters which were assessed. There were significantly more artifacts in sagittal T1-weighted sequences of the cervicothoracic and the thoracolumbar spine and in axial T2-weighted sequences of the thoracolumbar spine when obtained with the open MR scanner. Relevant artifacts were mainly due to motion for both, short-bore and open MR imaging. However, none of the examinations showed severe artifacts limiting their diagnostic value. Subgroup analyses excluding patients who underwent imaging after cross-referral revealed equally distributed results (*P*<0.0001, data not shown). Analysis of only those examinations which were performed with sedation also revealed higher qualitative image quality ratings for short-bore MR, but significant differences were only found for axial sequences (data not shown). Figures 2 to 5 show examples of MR images representing the respective range of qualitative image quality for the two scanners.

**Table 3:** Qualitative Image Quality Parameters.

	Cervicothoracic Spine			Thoracolumbar Spine		
	Short-Bore MR Imaging	Open MR Imaging	*P* Value	Short-Bore MR Imaging	Open MR Imaging	*P* Value
	Arithmetic mean (SD)			Arithmetic mean (SD)		
**T2w sagittal**	n = 18	n = 21		n = 30	n = 34	
Overall image quality	2.1 (1.1)	3 (1)	.006	1.8 (0.7)	2.7 (0.7)	<.0001
Contrast	1.8 (0.9)	2.8 (0.8)	.003	1.7 (0.7)	2.4 (0.7)	<.0001
Contour sharpness	2.2 (1)	2.9 (0.9)	.03	1.7 (0.7)	2.2 (0.6)	.002
Noise	1.7 (0.6)	32 (0.8)	<.0001	2.2 (0.6)	3.2 (0.7)	<.0001
Artifacts^a^	2.6 (1)	2.2 (0.9)	.25	2.1 (0.7)	2.3 (0.6)	.11
**T1w sagittal**	n = 18	n = 21		n = 30	n = 34	
Overall image quality	1.8 (0.8)	3.5 (0.7)	<.0001	1.8 (0.6)	2.7 (0.6)	<.0001
Contrast	1.9 (0.8)	3.2 (0.7)	<.0001	2 (0.7)	2.7 (0.6)	<.0001
Contour sharpness	2.2 (0.9)	3.8 (0.5)	<.0001	1.9 (0.6)	2.7 (0.6)	<.0001
Noise	1.6 (0.6)	2.7 (1)	.001	1.6 (0.5)	2.5 (0.6)	<.0001
Artifacts^a^	1.7 (0.8)	3 (1)	<.0001	1.6 (0.7)	2.6 (0.7)	<.0001
**T2w axial**	n = 19	n = 22		n = 49	n = 54	
Overall image quality	2.1 (0.7)	3.4 (0.7)	<.0001	2 (0.7)	3.5 (0.6)	<.0001
Contrast	2.2 (0.7)	3.2 (0.7)	<.0001	2.1 (0.8)	3.3 (0.6)	<.0001
Contour sharpness	2.1 (0.7)	3.2 (0.7)	<.0001	1.8 (0.7)	3 (0.6)	<.0001
Noise	1.8 (0.5)	3.5 (0.6)	<.0001	2.2 (0.6)	3.9 (0.4)	<.0001
Artifacts	2.7 (0.6)	2.7 (1)	.9	2.1 (0.9)	3.1 (0.8)	<.0001

An optimal score is defined as 1 and a poor score as 4. No sequence was rated nondiagnostic (score of 5) for any qualitative parameter.

^a^ Artifacts were mainly due to motion for both, short-bore and open MR imaging.

### Quantitative Analysis

**Figure 6:**
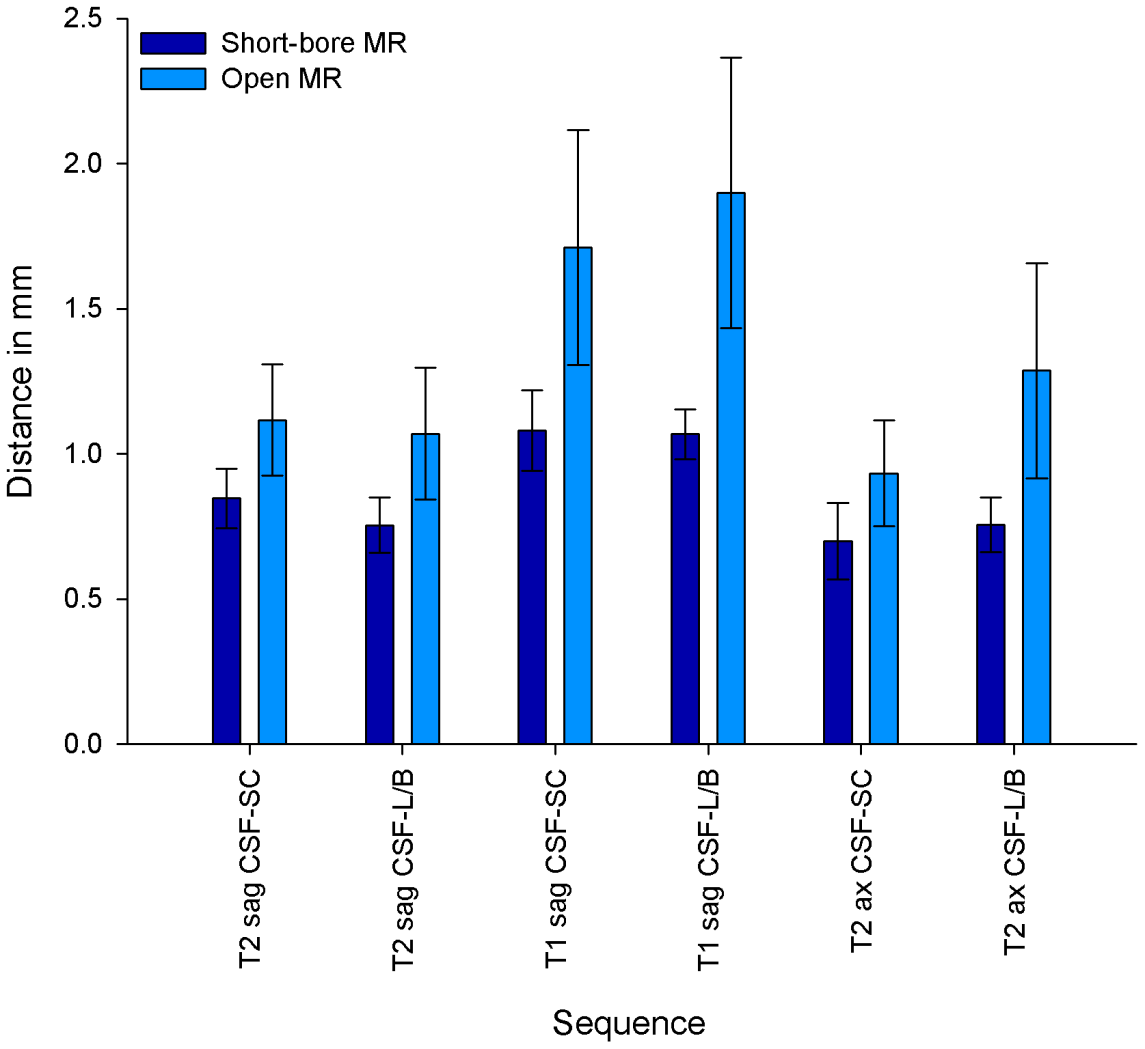
Contour Sharpness in MR images of the Cervicothoracic Spine. Contour sharpness as distance in mm (±SD) that is neeeded for the signal to increase from 25% to 75% of the grayscale pixel value profile obtained with imageJ (see Figure S1). Contour sharpness was defined for the interface between corticospinal fluid (CSF) and spinal cord (SC), and between corticospinal fluid and vertebral body (B)/posterior longitudinal ligament (L) in sagittal T2-weighted, sagittal T1-weighted and axial T2-weighted sequences. *P* values were <0.0001 for all assessed contours.

**Figure 7:**
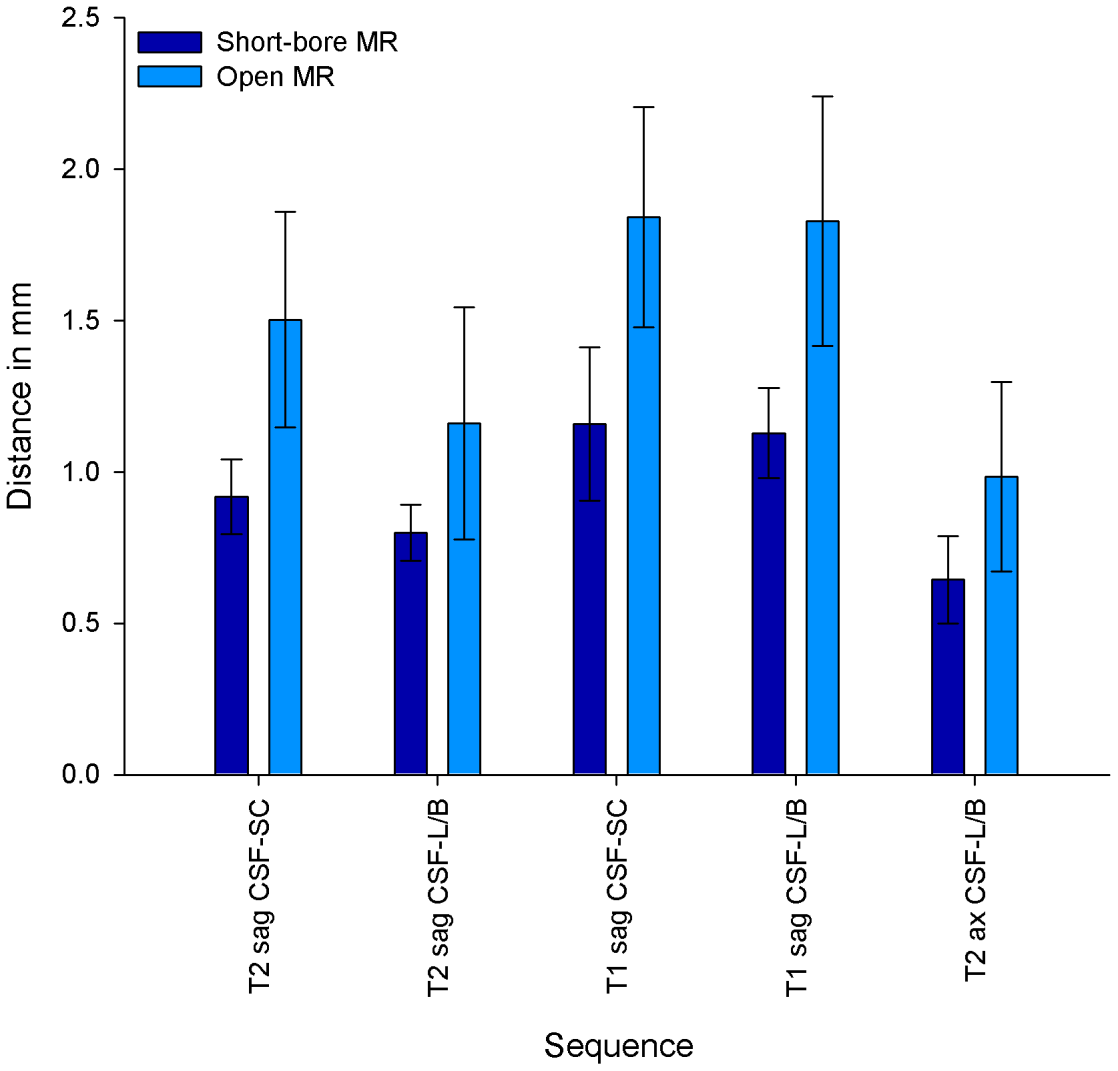
Contour sharpness in MR images of the thoracolumbar spine. Contour sharpness as distance in mm (±SD) that is neeeded for the signal to increase from 25% to 75% of the grayscale pixel value profile obtained with imageJ (see Figure S1). Contour sharpness was defined for the interface between corticospinal fluid (CSF) and spinal cord (SC), and between corticospinal fluid and vertebral body (B)/posterior longitudinal ligament (L) in sagittal T2-weighted, sagittal T1-weighted and axial T2-weighted sequences. In axial MR imaging of the thoracolumbar spine the spinal cord was normally not included and could thus not be assessed. *P* values were <0.0001 for all assessed contours.

Quantitative image quality parameters were in good agreement with the qualitative image quality results. The results computed separately for the different sequences of the cervicothoracic and the thoracolumbar spine are listed in Table 4. SNR was assessed for corticospinal fluid, spinal cord, vertebral bone, fat tissue, and muscle and an overall mean value was calculated. The mean SNR values of all available sequences were significantly higher for short-bore MR images than for open MR images (17.97 [SD, 6.58] versus 11.28 [SD, 4.35]; *P*<0.0001). The CNR values calculated were also higher for images obtained with the short-bore MR scanner (Table 4). Regarding quantitative assessment of contour sharpness, the mean values for the two assessed interfaces were significantly smaller in MR images obtained with the short-bore scanner than with the open MR scanner, thus indicating an improved contour sharpness (0.95 [SD, 0.24] versus 1.43 [SD, 0.48] and 0.83 [SD, 0.22] versus 1.32 [SD, 0.51]; *P*<0.0001). The detailed results of the contour sharpness measurement are also shown in Figures 6 and 7. Subgroup analyses of patients who underwent MR imaging without cross-referral and of sedated patients revealed similar results (data not shown).

**Table 4:** Quantitative Image Quality Parameters.

	Cervicothoracic Spine			Thoracolumbar Spine		
	Short-Bore MR Imaging	Open MR Imaging	*P* Value	Short-Bore MR Imaging	Open MR Imaging	*P* Value
	Arithmetic mean (SD)			Arithmetic mean (SD)		
**T2w sagittal**	n = 18	n = 21		n = 30	n = 34	
SNR^a^	16.9 (3.4)	9.23 (2.1)	<.0001	16.4 (3.7)	11.7 (2.8)	<.0001
CNR^b^						
Cerebrospinal fluid – Spinal cord	18.1 (11.6)	9.3 (5.5)	.005	22.3 (13.2)	14 (5.6)	.007
Cerebrospinal fluid – Vertebral bone	19.3 (10.3)	8.2 (5.7)	<.0001	24 (12.9)	11.7 (6.3)	<.0001
Contour sharpness^c^						
Cerebrospinal fluid – Spinal cord	0.8 (0.1)	1.1 (0.2)	<.0001	0.9 (0.1)	1.5 (0.4)	<.0001
Cerebrospinal fluid – Vertebral bone	0.8 (0.1)	1.1 (0.2)	<.0001	0.8 (0.1)	1.2 (0.4)	<.0001
**T1w sagittal**	n = 18	n = 22		n = 30	n = 34	
SNR^a^	22.9 (5.6)	14.4 (3.6)	<.0001	21.6 (4.4)	16.2 (3.4)	<.0001
CNR^b^						
Cerebrospinal fluid – Spinal cord	10.3 (11.5)	8.9 (5.1)	.6	17.7 (14.8)	9.4 (7)	.008
Cerebrospinal fluid – Vertebral bone	8.4 (6.1)	5.7 (3.8)	.1	8.2 (8.1)	3.9 (2.6)	.009
Contour sharpness^c^						
Cerebrospinal fluid – Spinal cord	1.1 (0.1)	1.7 (0.4)	<.0001	1.2 (0.3)	1.8 (0.4)	<.0001
Cerebrospinal fluid – Vertebral bone	1.1 (0.1)	1.9 (0.5)	<.0001	1.1 (0.1)	1.8 (0.4)	<.0001
**T2w axial** d	n = 19	n = 22		n = 49	n = 54	
SNR^a^	23.1 (6.4)	11.9 (2.7)	<.0001	13.2 (6.6)	7.3 (2.6)	<.0001
CNR^b^						
Cerebrospinal fluid – Spinal cord	11.9 (13.3)	11.1 (8.6)	.8			
White matter – Gray matter of the spinal cord	10.9 (12.7)	7.2 (4.7)	.2			
Cerebrospinal fluid – Vertebral bone	23.1 (14.2)	17.2 (10.9)	.1	15.2 (27.8)	8.9 (4.9)	.1
Contour sharpness^c^						
Cerebrospinal fluid – Spinal cord	0.7 (0.1)	0.9 (0.2)	<.0001			
Cerebrospinal fluid – Vertebral bone	0.8 (0.1)	1.3 (0.4)	<.0001	0.6 (0.1)	1(0.3)	<.0001

^a^ Overall mean value of signal-to-noise ratios (SNR) for corticospinal fluid, spinal cord, vertebral bone, fat tissue, and muscle.

^b^ Contrast-to-noise ratios (CNR) were calculated by substracting the respective SNR.

^c^ Values are distances in mm (±SD) that are neeeded for the signal to increase from 25% to 75% of the grayscale pixel value profile obtained with imageJ.

^d^ In axial MR imaging of the thoracolumbar spine, the spinal cord was normally not included and could thus not be assessed.

## Discussion

In this randomized comparison of image quality of spinal MR images obtained on high-field horizontal open and short-bore scanners, qualitative and quantitative parameters were in good agreement and indicated an advantage of short-bore MR imaging in all sequences. Short-bore MR images had a higher image quality with less image noise, higher contrast and contour sharpness, and higher SNR values than MR images obtained with the open MR scanner (Tables 3 and 4). CNR values were also significantly higher in short-bore MR images, except for T1-weighted sequences of the cervicothoracic spine and axial T2-weighted sequences (Table 4). In MR imaging of the cervical spine, one reason for these findings might be increased motion of swallowing and fluid in that region. However, the quantitative contour sharpness was not impaired in these sequences (Figures 6 and 7). Regarding the lower CNR values in T1-weighted sequences, an explanation could be the generally lower contrast in T1-weighted images compared to T2-weighted images in spinal imaging. Mean values of the quantitative contour sharpness measurement were higher in T1-weighted MR images of both MR scanners, thus indicating a decreased contour sharpness. The scan durations were significantly longer for MR imaging with the open MR scanner and there was a trend to higher perceived pain and noise levels during open MR imaging (Table 2, [8]). This might have caused the increased motion artifacts, which were found in part of the MR images obtained with the open scanner (Table 3).

The higher SNR, CNR, and quantitative contour sharpness which were achieved with the short-bore scanner may contribute to an increased sensitivity for the detection of pathologies. In our study, all examinations had diagnostic image quality. This might also be due to the appropriateness of all indications for MR imaging in this cohort [8], [19], [20]. However, the lower image quality achieved with open MR imaging may impede accurate diagnosis for indications requiring the highest possible resolution, e.g., diagnosis of spinal multiple sclerosis.

Image quality is influenced by several factors. One reason for the superior image quality with short-bore MR imaging found in our study is the higher field strength of the short-bore scanner (1.5 Tesla versus 1 Tesla). Stronger magnetization and higher precession rates at high field strengths increase SNR and CNR. In the 0.5 to 1.5 T range SNR and CNR theoretically increase linearly with field strength [24]. The differences in field strengths as well as gradient strengths were also the main reason for the longer scanning times in open MR imaging [8]. Another factor influencing image quality is homogeneity of the main magnetic field (B0). The horizontal magnetic field of the short-bore MR scanner runs around the z-axis of the body, while the vertical field lines in the open scanner run in anteroposterior direction and become more inhomogenous with increasing distance from the center of the magnet. Therefore, the vertical magnetic field is potentially more inhomogeneous, which can lead to nonuniformities in SI, and thus further decrease SNR and reduce subjective image quality. However, to our knowledge, there is no study addressing this issue and its potential impact on image quality in vertical field MR imaging. The typical homogeneity which is guaranteed by the vendors is for the Panorama 45×45×45 cm, 2.8 parts per million (ppm) and 0.4 ppm for the Avanto. The different coils which were used on the two scanners also influence image quality. The spine-array coils which are integrated in the short-bore MR table were normally closer to the region of interest, which resulted in increased SNR in that region. In vertical magnetic fields, solenoid coils have to be used. Of the coils which are available for the horizontal open scanner, the ST body/spine coil was the optimal choice for spinal MR imaging (personal communication with Dr. Bernhard Schnackenburg, Philips). Previous studies have shown superior performance of solenoid coils compared to surface coils, particularly regarding SNR [25], [26], [27]. Theoretically, SNR increases with the filling of a solenoid coil. However, subgroup analyses revealed no correlation of either SNR or image quality with patients' BMI.

To our knowledge, this is the first study to compare the image quality of two high-field MR scanners with specific patient-centered designs. Several other studies have compared the image quality of conventional closed MR scanners and found superior image quality for higher field strengths [28], [29], [30]. Others have compared high-field closed with low-field open MR scanners. Michel et al. found poor image quality in MR pelvimetry with a low-field open MR scanner [11]. Calabrese et al. concluded that open low-field contrast-enhanced MR imaging of the breast yielded good diagnostic performance in claustrophobic or oversized patients [31]. Mehdizade et al. found that diffusion-weighted MR imaging performed with a low-field open MR scanner was reliable for the evaluation of acute stroke [32]. Regarding the restrictions of conventional MR imaging, horizontal open MR scanners have shown potential for facilitating imaging of patients with claustrophobia or extreme obesity [5], [7], [8], and a better patient acceptance is assumed for open MR scanners [5], [10], [11]. Reduced claustrophobia rates have also been found with recent short-bore MR scanners [2], [9]. We have recently conducted a randomized controlled trial to compare horizontal open and short-bore MR scanners in patients at increased risk of claustrophobia during MR imaging and found a positive trend for open MR imaging [8]. However, there were claustrophobia rates of over 25% for both scanners. Thus, more patient-centered MR configurations are needed.

Strengths of our study include randomization and restriction of the anatomical regions examined to make the comparison as reliable as possible. We also used comparable sequence parameters on both MR scanners, which were kept constant in all examinations, as adjustment can affect signal intensities and contrast between tissues. Moreover, patient baseline characteristics were well matched between the two groups.

Our study has several limitations. First, in order to obtain comparable sequence parameters on both MR scanners, compromises had to be made regarding the best possible image quality still providing comparability. As the parameters were kept constant, they were also not optimized for single examinations. The second limitation with a possible impact on image quality was the use of different gradient coils on the two MR scanners. However, this cannot be avoided. Third, there were differences in the acquisition times due to the different field and gradient strengths. Theoretically, a longer scan time makes motion artifacts more likely, especially in patients who might suffer from pain or claustrophobia (Table 3). However, the differences in image quality we saw are not attributable to more motion artifacts degrading the quality of images acquired with the open MR scanner, because the image quality scores were higher for short-bore MR imaging in sequences with equal artifact ratings, and there were no severe artifacts in any sequence. Fourth, the viewing window-level parameters were not consistent in all images as this is not feasible in clinical practice. Window leveling was performed automatically with a constant approach leading to the best possible image appearance for diagnostic evaluation. Last, it should be mentioned that there are now high-field MR scanners with an even shorter and wider bore which have already shown to reduce the scan abortion rate in claustrophobic patients [9]. However, this improvement might come at the expense of image quality.

Future research should thus address image quality of MR scanners with a shorter and wider bore than the short-bore scanner which we used in our study. Moreover, further anatomical regions should be adressed regarding the comparison of high-field open versus short-bore MR imaging. This is particularly important because of the different demands on image quality depending on the medical indication and because of the different coils which are used for imaging of other anatomical regions. An intraindividual comparison could provide more insights into this issue and allow diagnostic comparison of the two scanners.

In conclusion, all examinations on both MR scanners were diagnostic, but qualitative and quantitative image quality parameters were rated higher for short-bore MR imaging. Most notable differences were found in overall image quality, mean SNR values, and quantitative contour sharpness. Previous studies have shown an advantage of open MR scanners regarding patient acceptance and imaging of claustrophobic or obese patients [5], [6], [7], [10], [11]. However, high claustrophobia rates have recently been found in patients at risk for both open and short-bore MR imaging [8]. Moreover, longer scanning times are required with recent open MR scanners. Thus, future developments should aim at designing more patient-centered MR scanners simultaneously providing high image quality without prolongation of scanning time.

## Supporting Information

Checklist S1 **CONSORT checklist.**
(DOC)

Protocol S1 Enders J, Zimmermann E, Rief M, Martus P, Klingebiel R, et al. (2011) Reduction of claustrophobia during magnetic resonance imaging: methods and design of the "CLAUSTRO" randomized controlled trial. BMC Med Imaging 11∶4.
(PDF)

Figure S1 **Contour Sharpness Measurement Using ImageJ.** A: In the example shown here a standardized line profile was drawn at a 90-degree angle over the contour of cerebrospinal fluid and spinal cord in a T1-weighted sagittal image of the cervicothoracic spine. The ROIs were drawn from the tissue with lower to the tissue with higher signal intensity. B: The grayscale pixel value profile was then calculated perpendicular to the axis of the line profile. C: The number of pixels (x-axis) that are neeeded for the signal to increase from 25% to 75% of the grayscale pixel value profile (colored section) was used as the measure of contour sharpness. Due to the different voxel sizes obtained with the two scanners the following formula was used to calculate the distance in mm: pixels measured in the grayscale pixel value profile x pixel length in mm.
(TIF)
