# Supplementary figures and images for: High-Field Open versus Short-Bore Magnetic Resonance Imaging of the Spine: A Randomized Controlled Comparison of Image Quality
Source: PLoS One. 2013 Dec 31;8(12):e83427. doi: 10.1371/journal.pone.0083427 (PMC3877023; doi:10.1371/journal.pone.0083427)

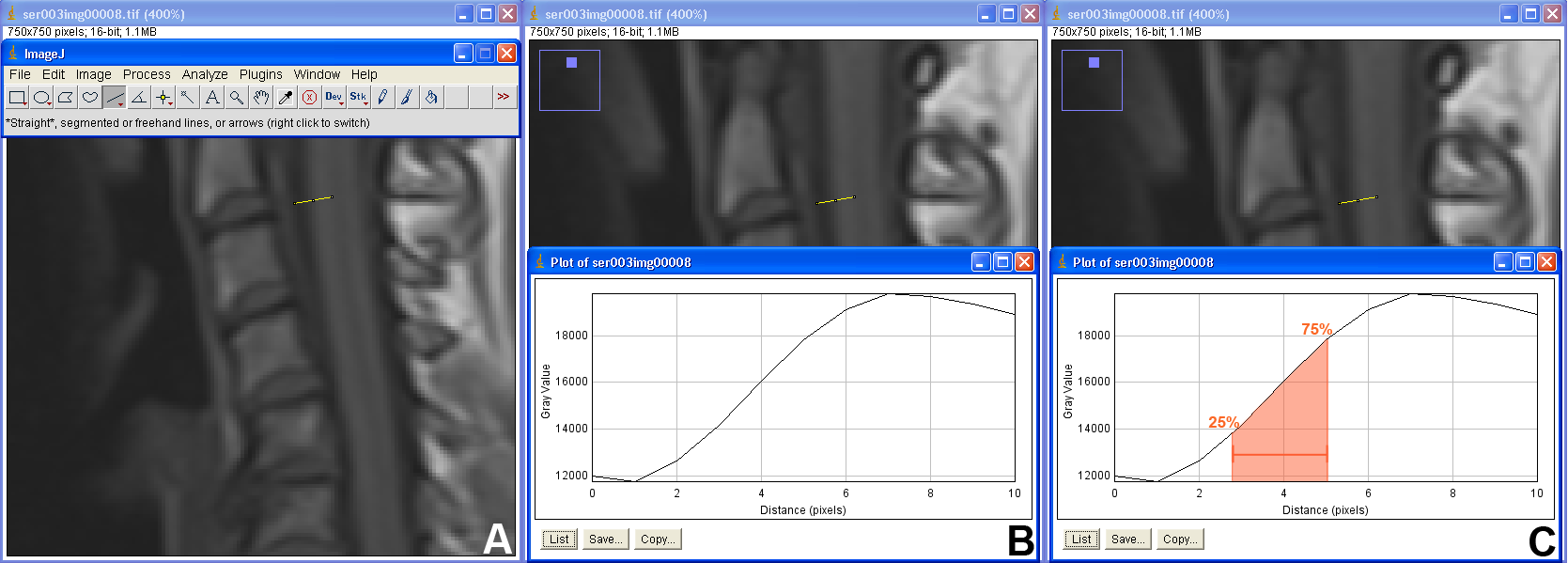

Supplement: Figure S1 — Contour Sharpness Measurement Using ImageJ. A: In the example shown here a standardized line profile was drawn at a 90-degree angle over the contour of cerebrospinal fluid and spinal cord in a T1-weighted sagittal image of the cervicothoracic spine. The ROIs were drawn from the tissue with lower to the tissue with higher signal intensity. B: The grayscale pixel value profile was then calculated perpendicular to the axis of the line profile. C: The number of pixels (x-axis) that are neeeded for the signal to increase from 25% to 75% of the grayscale pixel value profile (colored section) was used as the measure of contour sharpness. Due to the different voxel sizes obtained with the two scanners the following formula was used to calculate the distance in mm: pixels measured in the grayscale pixel value profile x pixel length in mm. (TIF) [file pone.0083427.s003.tif]
